# Clinical and Genetic Characterization of a Patient With SEC63-Related Autosomal Dominant Polycystic Liver Disease and an IFT140 Pathogenic Variant Associated With Polycystic Kidney Disease

**DOI:** 10.7759/cureus.105104

**Published:** 2026-03-12

**Authors:** Alan G Ortega-Macías, Udita Gupta, Tomas Escobar Gil, Jawairia Memon, Pablo R García

**Affiliations:** 1 Internal Medicine, University of New Mexico School of Medicine, Albuquerque, USA; 2 Gastroenterology and Hepatology, University of New Mexico School of Medicine, Albuquerque, USA; 3 Nephrology, University of New Mexico School of Medicine, Albuquerque, USA

**Keywords:** autosomal dominant polycystic kidney and liver disease, ciliopathy, genetic mutation, hepatic cysts, ift140, intraflagellar, kidney cysts, pathogenic variant, primary cilium, sec63

## Abstract

A 40-year-old female with a family history of polycystic kidney disease presented for evaluation. She was originally diagnosed with liver cysts in 2015 following an emergency department visit for suspected cyst rupture. Laboratory studies demonstrated preserved renal and hepatic function, with a creatinine level of 0.65 mg/dL and an estimated glomerular filtration rate (eGFR) of 114 mL/min/1.73 m². Abdominal MRI revealed numerous hepatic cysts, the largest measuring 12.9 × 10.8 cm, along with multiple bilateral renal cysts. The largest renal cyst measured 5.2 cm and was haemorrhagic and exophytic. Genetic testing identified heterozygous pathogenic variants in both SEC63 and IFT140. The patient is currently managed with serial imaging surveillance for hepatic cyst burden, portal hypertension, and total kidney volume.

This case illustrates a rare double-hit genetic entity. While SEC63 mutations rarely involve the kidneys, the co-existence of an IFT140 variant likely contributed to the development of bilateral renal cysts. This report emphasizes the role of comprehensive genetic testing in atypical polycystic presentations and highlights the importance of multidisciplinary monitoring in complex ciliopathies. Informed consent was obtained for publication of this case report.

## Introduction

SEC63 mutations cause isolated autosomal dominant polycystic liver disease (ADPLD) [1]. Conversely, pathogenic variants in IFT140, a gene involved in primary cilia function, represent a rare cause of autosomal dominant polycystic kidney disease (ADPKD) [1].

SEC63-related ADPLD is a rare genetic disorder that affects women more frequently than men, with an estimated global prevalence of less than 1 in 10,000 individuals. In contrast, ADPKD affects approximately 1 in 400 to 1,000 individuals worldwide. Pathogenic variants in IFT140 account for only about 2% of ADPKD cases [1]. The incidence of patients harbouring both IFT140 and SEC63 pathogenic variants who develop symptomatic hepatic and/or renal disease remains unknown and is poorly described in the medical literature [2-4].

Cyst formation in these disorders arises from dysfunction of the primary cilium, a solitary sensory and signalling organelle present on cholangiocytes and renal tubular epithelial cells. Primary cilia coordinate multiple signalling pathways, including calcium signalling, cyclic adenosine monophosphate (AMP), Wnt/β-catenin, and cell polarity pathways, which regulate epithelial proliferation, differentiation, and fluid transport. Mutations affecting ciliary structure or trafficking, such as those involving SEC63 and IFT140, impair proper ciliary assembly and function and disrupt these signalling networks. SEC63 variants may alter protein processing and ciliary signalling in cholangiocytes, contributing to hepatic cystogenesis. In contrast, IFT140 variants disrupt retrograde intraflagellar transport, leading to defective cilium maintenance and aberrant signalling in renal epithelial cells. These abnormalities promote unchecked epithelial proliferation, altered fluid secretion, and progressive cyst expansion in both liver and kidney tissues [1-4].

Although ciliary dysfunction is central to these ciliopathies, the clinical phenotype resulting from concurrent pathogenic variants in both SEC63 and IFT140 remains poorly characterised. This gap limits our understanding of the combined impact of these mutations on multi-organ cystogenesis. This case report therefore aims to clarify the mechanistic and clinical features of this dual-gene ciliopathy constellation and to provide additional diagnostic and pathophysiological insight into complex cystic liver-kidney disorders [1-4].

## Case presentation

A 40-year-old female with multiple hepatic cysts, along with a family history of a father with unspecified polycystic kidney disease, was initially diagnosed with liver cysts in 2015 after an ED visit for projectile vomiting. She was found to be vitally stable, and physical examination was remarkable only for right upper quadrant pain on deep palpation. Imaging reports from an outside hospital showed multiple liver cysts, with findings concerning for cyst rupture.

She presented 10 years later to the University of New Mexico nephrology clinic. Laboratory evaluation revealed normal renal and liver function: creatinine 0.65 mg/dL (estimated glomerular filtration rate (eGFR) 114 mL/min/1.73 m2), aspartate aminotransferase (AST) 25 U/L, alanine aminotransferase (ALT) 21 U/L, alkaline phosphatase (ALP) 71 U/L, total bilirubin 0.3 mg/dL, and albumin 4 g/dL (Table 1).

**Table 1:** Laboratory findings and reference ranges. eGFR: Estimated glomerular filtration rate; AST: Aspartate aminotransferase; ALT: Alanine aminotransferase; ALP: Alkaline phosphatase. Units: mg/dL: Milligrams per deciliter; g/dL: Grams per deciliter; mL/min/1.73 m²: Milliliters per minute per 1.73 m²; U/L: Units per liter.

Test	Value	Units	Reference range	Interpretation
Creatinine	0.65	mg/dL	0.6-1.2	Within normal limits
eGFR	114	mL/min/1.73 m²	>60	Within normal limits
AST	25	U/L	8-40	Within normal limits
ALT	21	U/L	10-40	Within normal limits
ALP	71	U/L	25-100	Within normal limits
Total bilirubin	0.3	mg/dL	0.1-1.0	Within normal limits
Albumin	4	g/dL	3.5-5.5	Within normal limits

**Figure 1:**
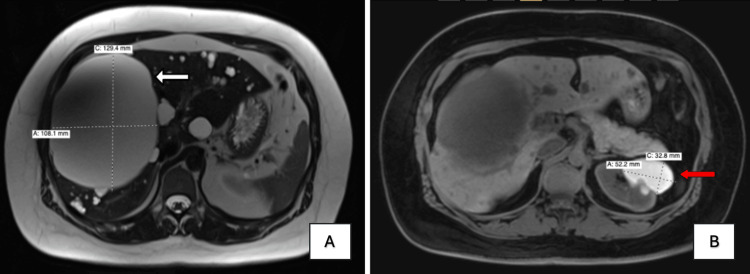
T2-weighted MRI of the abdomen with and without contrast showing hepatic and renal cysts. The white arrow in Figure 1A indicates a 12 × 10 cm hepatic cyst. The red arrow in Figure 1B indicates a 5 × 3 cm left renal cyst.

Abdominal MRI with and without contrast (Figure 1) demonstrated a mildly enlarged liver without steatosis or iron deposition. Numerous lobulated T2-hyperintense hepatic cysts of varying sizes were identified, the largest measuring 12.9 × 10.8 cm in the right hepatic lobe (Figure 1A). Multiple bilateral renal cysts were also present, with the largest measuring 5.2 × 3.3 cm as an exophytic cyst in the superior pole of the left kidney containing hemorrhagic or proteinaceous material (Figure 1B). No hydronephrosis was observed. Brain MRI showed no evidence of intracranial arterial aneurysms.

Genetic testing, performed via saliva swab, identified a heterozygous pathogenic/likely pathogenic variant in SEC63, consistent with ADPLD type 2. In addition, a pathogenic variant in IFT140 was detected, associated with ADPKD and IFT140-related recessive ciliopathies (Table 2). Due to preserved renal function, it was decided to defer starting tolvaptan until further kidney size assessment. The patient agreed to follow up with gastroenterology, with ultrasound surveillance every six months for evaluation of portal hypertension.

**Table 2:** Genetic testing results, associated conditions, and variant classifications. Variants were classified according to ACMG/AMP standards for sequence variant interpretation, which integrate population data, computational evidence, functional studies, segregation, and other lines of evidence into five categories: pathogenic, likely pathogenic, uncertain significance, likely benign, and benign. ADPKD: Autosomal dominant polycystic kidney disease: ACMG/AMP: American College of Medical Genetics and Genomics/Association for Molecular Pathology.

Gene	Condition(s)	Inheritance	Variant	Zygosity	Classification
SEC63	Polycystic liver disease 2	Autosomal dominant	c.715C>T (p.Arg239*)	Heterozygous	Pathogenic
IFT140	ADPKD-IFT140; IFT140-related recessive ciliopathies	Autosomal dominant and autosomal recessive	c.634G>A (p.Gly212Arg)	Heterozygous	Pathogenic

The diagnosis of ADPKD was established using well-validated age-specific imaging criteria; for adults with a positive family history, ≥3 renal cysts (unilateral or bilateral) at ages 15-39 years, and ≥2 cysts in each kidney at ages 40-59 years on ultrasound, are diagnostic, while MRI criteria include >10 total renal cysts for ages 16-40, and genetic testing confirmed the suspected diagnosis [5]. Similarly, a formal diagnosis of ADPLD was established based on the patient meeting validated criteria: the presence of a pathogenic ADPLD gene mutation (SEC63) and hepatic imaging meeting cyst number thresholds, namely ≥1 liver cyst in individuals <40 years and ≥4 liver cysts in individuals ≥40 years [5].

## Discussion

The clinical presentation of polycystic liver disease (PLD) is genetically heterogeneous and most commonly involves mutations in genes such as PRKCSH and SEC63. The SEC63 gene encodes a protein localised to the endoplasmic reticulum membrane that is essential for protein translocation and quality control. Although SEC63 mutations are the second most common cause of isolated ADPLD, they are rarely associated with significant renal involvement [4-5]. In this case, the patient exhibited a more complex phenotype characterised by both massive hepatic cysts and bilateral renal cysts. This deviation from the typical liver-only SEC63 phenotype suggests that the presence of a second genetic hit, specifically the IFT140 variant, altered the clinical trajectory of the disease.

The IFT140 gene encodes a component of intraflagellar transport complex A, which is critical for the assembly and maintenance of the primary cilium. Although IFT140 is frequently associated with syndromic ciliopathies, recent evidence has identified it as a rare cause of a mild ADPKD phenotype. The two-hit hypothesis in ciliopathies suggests that the overall functional threshold of ciliary proteins influences the severity of cyst formation. In this patient, a synergistic effect is suspected, in which the SEC63 mutation likely drove hepatic cystogenesis, while the IFT140 variant provided the additional genetic hit necessary to trigger the development of bilateral renal cysts. This dual-mutant profile complicates the traditional classification of ADPLD and ADPKD, placing this patient in a unique intermediate category [5-8].

Management of this rare genetic entity requires a multidisciplinary approach focused on both hepatic and renal surveillance. Although the patient’s eGFR remains preserved, the presence of an IFT140 variant and a 5.2 cm haemorrhagic renal cyst necessitates long-term monitoring of total kidney volume (TKV) to assess the risk of progression to chronic kidney disease. Concurrently, the large hepatic cysts, measuring up to 12.9 cm, pose risks of rupture, mass effect, and portal hypertension. Therapeutic interventions such as somatostatin analogues or aspiration sclerotherapy may be considered if symptoms such as projectile vomiting recur. Ultimately, this case underscores the utility of broad-panel genetic testing in identifying complex genotypes that do not conform to standard clinical phenotypes [9-10].

Concurrently, the large size of the hepatic cysts (12.9 cm) poses risks of rupture, mass effect, and portal hypertension. Therapeutic interventions, such as somatostatin analogues or aspiration sclerotherapy, may be considered if symptoms such as the patient’s initial projectile vomiting recur. Ultimately, this case underscores the utility of broad-panel genetic testing in identifying complex genotypes that do not fit standard clinical phenotypes [10-13].

Integrating the Mayo Classification, which categorizes patients into Classes 1A through 1E based on age-adjusted height-normalized TKV (htTKV), would be essential for predicting the future rate of eGFR decline. However, while this classification is the gold standard for typical ADPKD (Class 1A-1E), the patient’s imaging demonstrates an atypical pattern (Mayo Class 2) due to the presence of a dominant exophytic haemorrhagic cyst (5.2 cm), rather than the diffuse, uniform cyst distribution seen in classic PKD1/2 mutations. This classification further supports the unique genetic synergy between SEC63 and IFT140, where renal involvement remains segmental rather than globally proliferative [14].

## Conclusions

This case report illustrates a rare and complex clinical entity that expands the known phenotypic spectrum of polycystic diseases. Typically, SEC63-related ADPLD is characterised by a “liver-only” phenotype, with kidney cysts being an infrequent finding that rarely progresses to significant renal impairment. In this patient, however, the presence of a concomitant IFT140 variant was associated with a distinct combined hepatorenal phenotype, featuring both hepatic cystogenesis and bilateral renal cysts.

While the co-occurrence of these variants is consistent with current “total functional threshold” models of ciliopathies, this single-case observation demonstrates a clinical association rather than a confirmed mechanistic interaction. Without functional validation or segregation analysis, the precise nature of the genetic relationship, whether synergistic or additive, remains interpretive. Nonetheless, the case underscores the utility of broad-panel genetic testing and standardised imaging metrics, such as the Mayo Clinic Imaging Classification, in characterising atypical cystic presentations. Integrating these tools is important for the longitudinal surveillance of TKV and the assessment of renal risk in patients harbouring rare or dual-genotype profiles.

## References

[1] Senum SR, Li YS, Benson KA (2022). Monoallelic IFT140 pathogenic variants are an important cause of the autosomal dominant polycystic kidney-spectrum phenotype. Am J Hum Genet.

[2] Waanders E, Venselaar H, te Morsche RH (2010). Secondary and tertiary structure modeling reveals effects of novel mutations in polycystic liver disease genes PRKCSH and SEC63. Clin Genet.

[3] Besse W, Dong K, Choi J (2017). Isolated polycystic liver disease genes define effectors of polycystin-1 function. J Clin Invest.

[4] Waanders E, te Morsche RH, de Man RA, Jansen JB, Drenth JP (2006). Extensive mutational analysis of PRKCSH and SEC63 broadens the spectrum of polycystic liver disease. Hum Mutat.

[5] Zhang ZY, Wang ZM, Huang Y (2020). Polycystic liver disease: classification, diagnosis, treatment process, and clinical management. World J Hepatol.

[6] Davila S, Furu L, Gharavi AG (2004). Mutations in SEC63 cause autosomal dominant polycystic liver disease. Nat Genet.

[7] Dordoni C, Zeni L, Toso D (2024). Monoallelic pathogenic IFT140 variants are a common cause of autosomal dominant polycystic kidney disease-spectrum phenotype. Clin Kidney J.

[8] Stein Q, Westemeyer M, Darwish T (2023). Genetic counseling in kidney disease: a perspective. Kidney Med.

[9] van Aerts RM, van de Laarschot LF, Banales JM, Drenth JP (2018). Clinical management of polycystic liver disease. J Hepatol.

[10] Olaizola P, Rodrigues PM, Caballero-Camino FJ (2022). Genetics, pathobiology and therapeutic opportunities of polycystic liver disease. Nat Rev Gastroenterol Hepatol.

[11] Chapman AB, Devuyst O, Eckardt KU (2015). Autosomal-dominant polycystic kidney disease (ADPKD): executive summary from a Kidney Disease: Improving Global Outcomes (KDIGO) Controversies Conference. Kidney Int.

[12] Wijnands TF, Görtjes AP, Gevers TJ (2017). Efficacy and safety of aspiration sclerotherapy of simple hepatic cysts: a systematic review. AJR Am J Roentgenol.

[13] Guler S, Cimen S, Hurton S, Molinari M (2015). Diagnosis and treatment modalities of symptomatic polycystic kidney disease. Polycystic Kidney Disease.

[14] Irazabal MV, Rangel LJ, Bergstralh EJ (2015). Imaging classification of autosomal dominant polycystic kidney disease: a simple model for selecting patients for clinical trials. J Am Soc Nephrol.

